# Image Clustering with Optimization Algorithms and Color Space

**DOI:** 10.3390/e20040296

**Published:** 2018-04-18

**Authors:** Taymaz Rahkar Farshi, Recep Demirci, Mohammad-Reza Feizi-Derakhshi

**Affiliations:** 1 Computer Engineering Department, Technology Faculty, Gazi University, Ankara 06500, Turkey; 2 Department of Computer Engineering, University of Tabriz, Tabriz 51666, Iran

**Keywords:** image clustering, color space, thresholding

## Abstract

In image clustering, it is desired that pixels assigned in the same class must be the same or similar. In other words, the homogeneity of a cluster must be high. In gray scale image segmentation, the specified goal is achieved by increasing the number of thresholds. However, the determination of multiple thresholds is a typical issue. Moreover, the conventional thresholding algorithms could not be used in color image segmentation. In this study, a new color image clustering algorithm with multilevel thresholding has been presented and, it has been shown how to use the multilevel thresholding techniques for color image clustering. Thus, initially, threshold selection techniques such as the Otsu and Kapur methods were employed for each color channel separately. The objective functions of both approaches have been integrated with the forest optimization algorithm (FOA) and particle swarm optimization (PSO) algorithm. In the next stage, thresholds determined by optimization algorithms were used to divide color space into small cubes or prisms. Each sub-cube or prism created in the color space was evaluated as a cluster. As the volume of prisms affects the homogeneity of the clusters created, multiple thresholds were employed to reduce the sizes of the sub-cubes. The performance of the proposed method was tested with different images. It was observed that the results obtained were more efficient than conventional methods.

## 1. Introduction

The classification of pixels in images is one of the most difficult tasks in image processing because at the end of the procedure, it is desired that pixels assigned into a cluster must be the same or quite similar and must be different from pixels in other clusters. Moreover, pixels assigned into the same cluster must be similar in terms of spatial coordinates. In the literature, several methods were proposed to solve the problem. It is claimed that some of these methods result in more accurate segmentation, whereas some claim that they do faster segmentation than others. Fundamentally, the critical questions to be answered are about the number of clusters for any gray scale or color image and computation cost of the algorithm.

Common image clustering algorithms based on data classification methods are K-means [1] and fuzzy c-means (FCM) [2]. These techniques are successful in the clustering of images that have a certain number of clusters. Nevertheless, if the number of clusters is not known, which is typical for the segmentation process, clustering is not possible [3]. Furthermore, these algorithms are iterative and pixels are used with the algorithms more than once. Additionally, the centers of the clusters are set randomly and the clustering procedure needs to be repeated more than once to reach correct results [4]. As the number of iterations increases, the time consumed by the procedure grows [5]. The computational complexity of the FCM algorithm is O(**ncl**) where **n**, c and **l** is the number of pixels, clusters, and iteration, respectively [6]. 

Apart from K-means and FCM methods, region-based segmentation techniques are based on finding adjacent pixels with similar features [7]. Thus, the similarity judgment of pixels must be appropriately evaluated so that consistent regions can be produced. A segmentation map starting with small regions, known as seeds, is generated [8]. Accordingly, neighboring pixels are evaluated to grow the seeds to obtain the regions. If any pixel is sufficiently similar to an adjacent region, the pixel is included in the region. The seed regions are created either automatically or selected by the user. Automatic creation of these seeds brings an additional computational cost.

For gray scale images, binarization and multilevel thresholding are well-known clustering approaches. They are based on the processing of histogram information rather than spatial pixel similarity computations so that the optimal thresholds are obtained. Consequently, histogram-based approaches are faster than FCM and they yield reasonable results. Nevertheless, there have been difficulties in the integration of multilevel thresholding techniques into color image segmentation so far.

Entropy is one of the most famous methods in dealing with image processing problems [9]. In this regard, Kapur’s entropy is frequently used in image segmentation. Furthermore, Otsu thresholding is one of the well-known image segmentation schemes. However, the inefficient formulation of the time cost of the Otsu and Kapur algorithms makes these methods impractical, especially in the selection of multilevel thresholds. Even though extensive efforts have been made to achieve image segmentation, autonomic techniques that work in real-time still remain a challenge [10]. To overcome this problem, researchers used optimization algorithms to realize Otsu’s and Kapur’s criteria [11,12,13,14,15,16,17,18]. Horng and Jiang [19] proposed a firefly optimization algorithm for the maximum entropy criterion, while Maitra and Chatterjee [20] proposed a hybrid cooperative in-depth learning model by using a particle swarm optimization algorithm to achieve the maximum entropy criterion. Moreover, Sathya and Kayalvizhi [21] introduced a bacterial foraging algorithm to optimize the maximum entropy criterion and Otsu’s minimum variance criteria. In addition to these studies, Sathya and Kayalvizhi [22] proposed a modified bacterial foraging algorithm for the maximum entropy and minimum variance criterions. Oliva et al. [23] proposed a multilevel image thresholding based on the harmony search optimization algorithm for the related approaches. Lastly, Horng [24] applied the artificial bee colony (ABC) algorithm to optimize the maximum entropy criterion. 

In this study, a novel approach for clustering color images by using multilevel thresholding has been proposed. The application of binarization methods for color image clustering has recently been introduced by Demirci et al. [8], where a single threshold for each color channel was computed by means of the Otsu and Kapur methods. Then, color space was partitioned into eight sub-cubes where the pixels within each cube were assigned to the same cluster. Although the method was quite fast, clustering performance was not adequate for some color images, as only eight clusters were assigned. In order to eliminate the drawback of the algorithm and increase the number of clusters, multilevel thresholding for each channel has been suggested. The threshold values for each color channel were separately estimated by particle swarm optimization (PSO) and the forest optimization algorithm (FOA) [25]. Accordingly, the volume of each sub-cube was decreased, and clustering performance in terms of region homogeneity was improved.

## 2. Multilevel Thresholding and Color Space Partition

In gray scale images, the gray levels are in the range of {0,1,2,…,L−1}. Thus, the probability of the *i*th gray level could be defined as

(1) $$p_{i}=h_{i}/(M\times N)$$

where *M* and *N* are dimensions of image, $h_{i}$ denotes the number of pixels corresponding to gray level *i*, 0≤i≤(L−1). When multiple thresholds such as *t*_1_, *t*_2_,…, *t_m_* are used for classification, the number of clusters created will be *m* + 1. Accordingly, gray level intervals of clusters formed will be *c*_0_ → [0, …, t1 − 1], *c*_1_ → [*t*_1_, …, *t*_2_ − 1] and *c_m_* → [*t_m_*, …, L − 1]. The critical issue in multilevel thresholding is the determination of thresholds. One common approach is to define a multi-variable objective function in terms of *t*_1_, *t*_2_,…, *t_m_*. The optimal thresholds are then obtained by maximizing the objective function. The most frequently preferred thresholding functions are Kapur’s entropy criterion approach [26] and Otsu’s between-class variance technique [27]. Although the initial proposals of the Otsu and Kapur algorithms were for binary thresholding, the extension for multilevel thresholding is also possible [21]. Consequently, Kapur’s entropy criterion is extended for multilevel thresholding by maximizing the objective function stated as:

(2) $$J(t_{1},t_{2},\ldots ,t_{m})=H_{0}+H_{1}+H_{2}+\ldots +H_{m}$$

where

$$H_{0}=-\sum_{i=0}^{t_{1}-1}\frac{p_{i}}{\omega_{0}}\ln\frac{p_{i}}{\omega_{0}},\omega_{0}=\sum_{i=0}^{t_{1}-1}p_{i}\;H_{1}=-\sum_{i=t_{1}}^{t_{2}-1}\frac{p_{i}}{\omega_{1}}\ln\frac{p_{i}}{\omega_{1}},\omega_{1}=\sum_{i=t_{1}}^{t_{2}-1}p_{i}\;H_{2}=-\sum_{i=t_{2}}^{t_{3}-1}\frac{p_{i}}{\omega_{2}}\ln\frac{p_{i}}{\omega_{2}},\omega_{2}=\sum_{i=t_{2}}^{t_{3}-1}p_{i}\;\vdots \;H_{m}=-\sum_{i=t_{m}}^{L-1}\frac{p_{i}}{\omega_{m}}\ln\frac{p_{i}}{\omega_{m}},\omega_{m}=\sum_{i=t_{m}}^{L-1}p_{i}$$

where *H*_0_ to *H_m_* are partial entropies of the histogram, and $\omega_{0}$ to $\omega_{m}$ are partial probabilities of the histogram. The threshold values: *t*_1_…*t_m_* are the gray levels that maximize the objective function given in Equation (2). Additionally, the multilevel thresholds for any gray scale image could also be estimated by means of Otsu’s between-class variance algorithm. Thus, a *m*-dimensional objective function is described as follows:

(3) $$J(t_{1},t_{2},\ldots ,t_{m})=\sigma_{0}+\sigma_{1}+\sigma_{2}+\ldots +\sigma_{m}$$

where

$$\sigma_{0}=\omega_{0}{\left(\mu_{0}-\mu_{T}\right)}^{2}\;\sigma_{1}=\omega_{1}{\left(\mu_{1}-\mu_{T}\right)}^{2}\;\sigma_{2}=\omega_{2}{\left(\mu_{2}-\mu_{T}\right)}^{2}\;\vdots \;\sigma_{m}=\omega_{m}{\left(\mu_{m}-\mu_{T}\right)}^{2}$$

with

$$\omega_{0}=\sum_{i=0}^{t_{1}-1}p_{i},\mu_{0}=\sum_{i=0}^{t_{1}-1}\frac{i\;p_{i}}{\omega_{0}}\;\omega_{1}=\sum_{i=t_{1}}^{t_{2}-1}p_{i},\mu_{1}=\sum_{i=t_{1}}^{t_{2}-1}\frac{i\;p_{i}}{\omega_{1}}\;\vdots \;\omega_{m}=\sum_{i=t_{m}}^{L-1}p_{i},\mu_{m}=\sum_{i=t_{m}}^{L-1}\frac{i\;p_{i}}{\omega_{m}}$$

and

$$\mu_{T}=\sum_{i=0}^{L-1}i\;p_{i}$$

where $\mu_{0}$ to $\mu_{m}$ are means of classes. Furthermore, $\mu_{T}$ is the mean of the image. In both cases, there are constraints that are defined as: $t_{1}<t_{2}<t_{3}<\ldots <t_{m}$. As can be seen in both methods, multilevel thresholding is a kind of multi-variable optimization problem to be solved. Although there have been various approaches for optimization, the most common and newest algorithms have been used in this study. The particle swarm optimization (PSO) algorithm suggested by Kenedy and Eberhart in 1990 is a widely used one [28]. It was inspired from the social behavior of birds and fish. Like other optimization algorithms, the locations of particles in the swarm are also randomly initialized. Next, in each iteration all particles are updated as follows:

(4) $$\begin{array}{l} v_{i}(t+1)=w\;v_{i}(t)+R_{1}C_{1}(p_{i}^{best}-x_{i})+R_{2}C_{2}(g^{best}-x_{i})\\ x_{i}(t+1)=x_{i}(t)+v_{i}(t+1) \end{array}$$

where $x_{i}$ and $v_{i}$ are the position and velocity of each particle, at iteration t, $R_{1}$ and $R_{2}$ are two random values between [0, 1], *w* is the inertia weight, both $C_{1}$ and $C_{2}$ are learning factors that control the influence of personal best and global best, respectively, $p_{i}^{best}$ responds to the best position that the *i*th particle has ever found. Finally, $g_{}^{best}$ is the best position found so far in the entire population.

On the other hand, one of the recently introduced optimization algorithms, called the forest optimization algorithm (FOA) and proposed by Ghaemi and Feizi-Derakhshi, is based on the surviving processes of trees in forest. It could be observed in the forest that some trees survive for several decades and continue to their generation while others live for a limited period. Therefore, some distinguished trees live for centuries since they are seeded in the geographically best places. By emulating the natural seed dispersal process, finding the distinguished trees in the forest is executed by the attempts performed by the FOA. Trees use different strategies of seeding in order to continue their generation. Seed dispersal methods consist of two different kinds: long-distance seed dispersal, and nature local seed dispersal. At the beginning of the seeding process in nature, some seeds fall and germinate just near the trees. This procedure is called ‘local seeding’. On the other hand, if seeds are transferred far away from trees by means of water, wind or even animals, this procedure is called long-distance seeding or ‘global seeding’. After the seeds descend on the land due to local or global seeding, the seeds germinate and turn into saplings. Yet, the germination of every seed and the chance of a seed becoming a tree in the forest is not possible.

**Figure 1:**
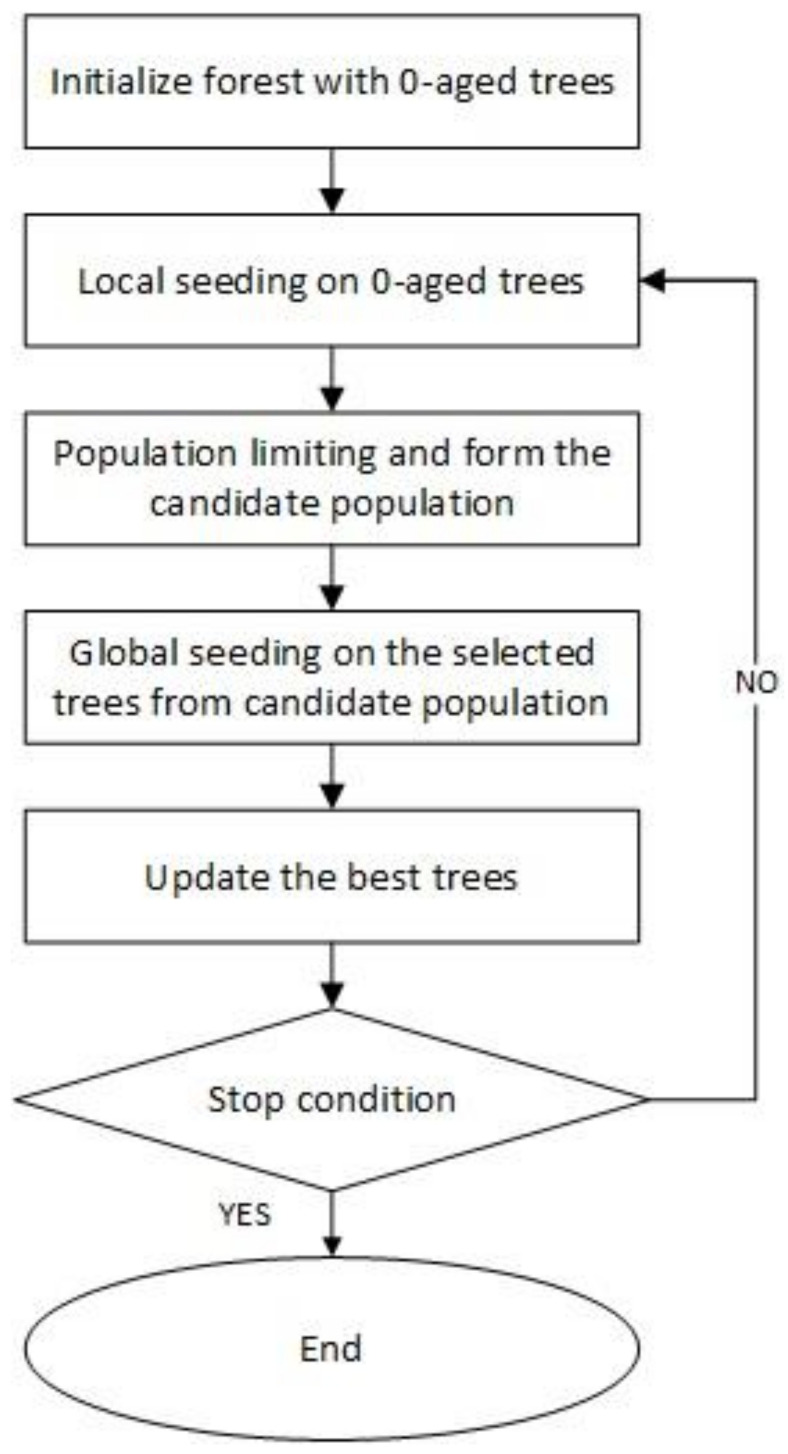
Flowchart of a forest optimization algorithm (FOA).

FOA consists of three steps: local seeding, population limiting, and global seeding. Figure 1 shows the flowchart of FOA [25]. Identifying a solution for the problem is represented by each tree FOA beginning with the initial random population, in a similar way to any other evolutionary algorithm. Each tree in the initial population has zero age and its age, except for the newly generated trees, is increased at each iteration step. A tree is allowed to live up to a maximum age, which is defined as the ‘life time’ parameter. If the age of a tree reaches to maximum age, it will be removed from the forest.

Some seeds disperse around the tree and germinate into young seedlings during the seeding process. These seedlings take part in a competition for the use of minerals, sun light, and other resources. Local seeding changes (also defined as “LSC”) is a stage when the number of variables’ values change during the local seeding stage. This stage is operated on zero-aged trees.

**Figure 2:**
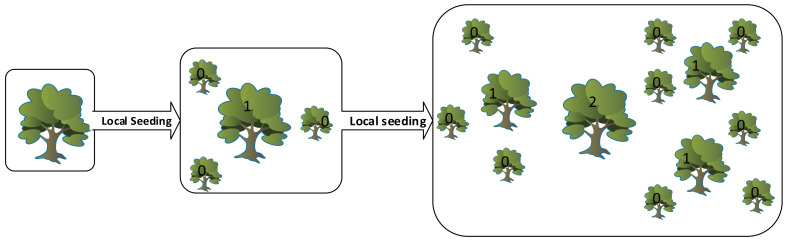
Two consequent iterations of the local seeding stage.

Figure 2 shows two consequent iterations of the local seeding stage. After the local seeding stage, in order to avoid endless expansion of the forest, a control method on the number of trees is considered. “Area Limit” and “Life Time” parameters are the two criteria considered to achieve this goal. Addition of the trees into the candidate population is performed only upon trees whose age is greater than lifetime. In addition, exceeding the area limit parameter of the trees, the additional trees are transferred to the candidate population as well.

As stated before, some seeds are carried from trees to faraway locations with the aid of natural events such as wind, flow of water, or animals. Subsequently, they will have more chance to survive. 

Global seeding simulates this process. “Transfer rate” is a stage in the global seeding stage that is used to set a percentage to the candidate population. The transfer rate is predefined and some of the trees from the candidate population are randomly selected according to transfer rate. 

**Figure 3:**
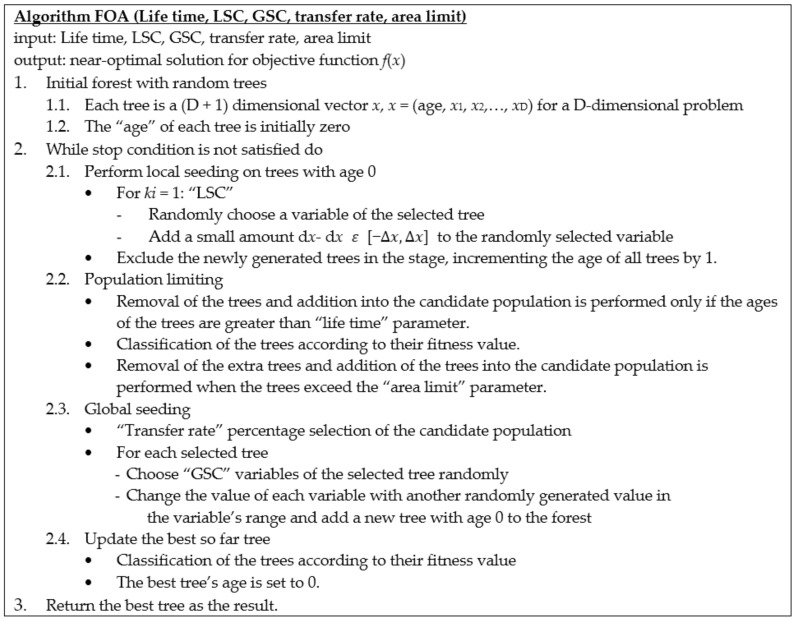
Pseudo code of FOA.

**Figure 4:**
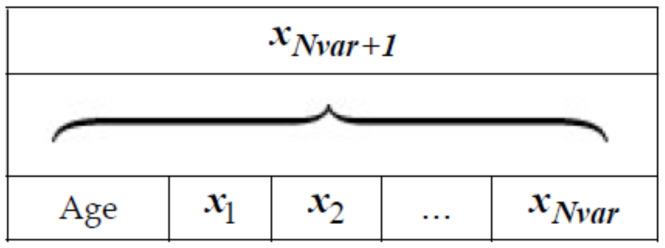
A solution representation of FOA.

In the variable range, some variables of the selected trees are chosen and the values are exchanged with other values. This procedure allows searching in the broad region of the search space. The trees that are newly generated are set to age zero and are added to the forest. The number of the selected variables for global seeding in each tree is defined as “Global seeding changes” or “GSC”. Figure 3 is the pseudo code of FOA. In order to simulate a problem using FOA, each potential solution is represented as Figure 4. If a problem has N_var_ dimension, each tree will have N_var+1_ variables with the “Age” part showing the age of the related trees. 

The multilevel thresholding in image processing is a multivariable optimization problem in which objective functions were defined in Equations (2) and (3). By considering Figure 4, the objective function based on Otsu’s approach and on Kapur’s entropy criterion, each solution with the Otsu and Kapur procedure was represented with a tree $J\left(H_{0},H_{1},H_{2}\cdots ,t_{m}\right)$ and $J\left(\sigma_{0},\sigma_{1},\sigma_{2}\cdots ,\sigma_{m}\right)$, respectively.

The stated optimization algorithms are employed to find the optimal threshold values on image histogram segmentation. The process of the proposed scheme is as follows:

**Step** **1.** Specify the lower and upper boundaries of the optimization algorithm to limit the minimum and maximum threshold value.
**Step** **2.** Initialize the *m*-dimension positions of each individual in the population. Dimension values correspond to the number of the threshold, such that *t*_1_ < *t*_2_ < *t*_3_ <…< *t_m_*.
**Step** **3.** Evaluation of each individual objective function among the population using Equations (2) and (3).
**Step** **4.** Updating the positions of threshold values for each individual.
**Step** **5.** If termination conditions are met, then stop. Otherwise go back to step 3.
**Step** **6.** Return the optimal threshold values corresponding to the global best individual.

**Figure 5:**
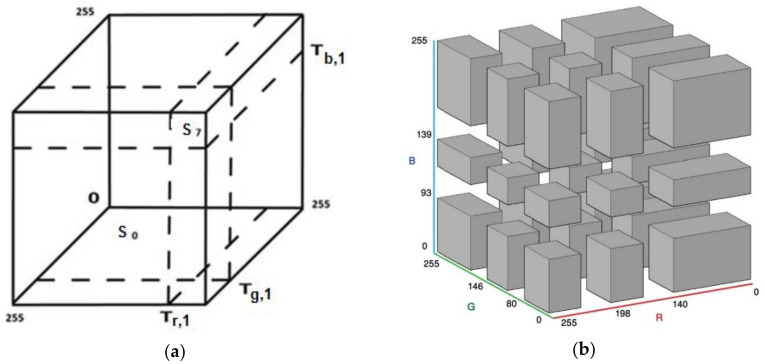
(**a**) 3D color space and assigned clusters, *m* = 1 (**b**) 3D color space and assigned clusters, *m* = 2.

Once multilevel thresholds have been obtained with the related optimization algorithms, they are integrated into the color space partition techniques proposed by Demirci et al. [8]. The color space partition algorithm was initially developed for a single threshold. However, in this study, it was extended by using a multilevel threshold. Therefore, it is appropriate to clarify the initial strategy. The binarization or multilevel clustering of gray scale images is performed by means of the threshold values obtained with the Otsu and Kapur methods. Nevertheless, color images consist of three channels and each color channel requires its own thresholds. Even if the thresholds for each channel are obtained, it is a critical issue to establish meaningful clusters with information coming from each color channel. In this study, the threshold values computed for each channel are used to create subsets of color space, as shown in Figure 5. In other words, the color cube is divided into sub-cubes or prisms. Then, each pixel in any of the sub-cubes or prisms is included in the same cluster. At the start, it may seem that unrelated clusters may be created with this approach. However, quite reasonable results in images have been obtained in experiments. When looking closely at Figure 5, it can be seen that the volume of each sub-cube or prism depends on the thresholds of each channel, and are not the same. Therefore, shapes of sub-cubes or prisms are related to the distributions of pixel intensity in the image. Consequently, a unique color space partition scheme for every particular image will be created. It is clear that the homogeneity of clusters with larger volumes will be low compared with that of clusters with small volumes. Nevertheless, it is not an unsolvable problem. If the volume of each cube could be reduced by increasing the number of thresholds (multilevel thresholding), the homogeneity will grow. Thus, the maximum number of clusters that could be created for a color image will be as follows:

(5) $$c_{m}={\left(m+1\right)}^{3}$$

When a single threshold for each channel, *m* = 1, is used, eight sub-clusters are established. If *m* = 2, *c_m_* will be 27; if *m* = 3, *c_m_* = 64, and so on. Despite Figure 5a showing a color space partition with a single threshold, it could be extended for multilevel thresholds. Cluster labels and codes for labels with binary numbers are given in Table 1. Furthermore, color space partition rules are described in Table 1. Figure 5b shows the sub-cubes or prisms obtained with two thresholds for the Lena image: T_r,1_, T_g,1_, T_b,1_ (140, 80, 95) and T_r,2_, T_g,2_, T_b,2_ (198, 145, 138). 

## 3. Experimental Results and Discussion

Although the classification of images with larger sizes take a long time with iterative methods such as K-means or fuzzy c-means (FCM) compared with that of images with small sizes, the computational cost of a developed algorithm is independent of image size, since clustering is performed by means of the single-dimensional histogram data of each channel, rather than pixel-wise calculations. As a result, pixels in images are employed only once to obtain histograms. The main computational effort is required to estimate the thresholds in terms of Kapur’s criteria and Otsu’s objective functions by using FOA and PSO. Therefore, performance comparisons were done with an FCM algorithm, which is a commonly used approach in image segmentation. Furthermore, in order to make a quantitative assessment of the achieved results, the image segmentation evolution function proposed by Liu and Yang [29,30] has been employed. This segmentation evolution function is defined as follows:

(6) $$F=\frac{1}{1000\left(M\times N\right)}\sqrt{R}\overset{R}{\underset{i=1}{\sum }}\frac{e_{i}^{2}}{\sqrt{A_{i}}}$$

where *M* × *N* represents the size of the image, *R* is the number of obtained regions. $A_{i}$ is the number of pixels in the *i*th region, $e_{i}$ is the average of color error in the *i*th region, which is defined as the sum of the Euclidean distances between pixels of the *i*th region in the original color image and the attributed pixel values of the *i*th region in the segmented image. The term $\sqrt{R}$ is used to prevent the *F*-value from shrinking excessively. As the number of regions increases, the value of *F* decreases. A small value for *F* is desired.

**Figure 6:**
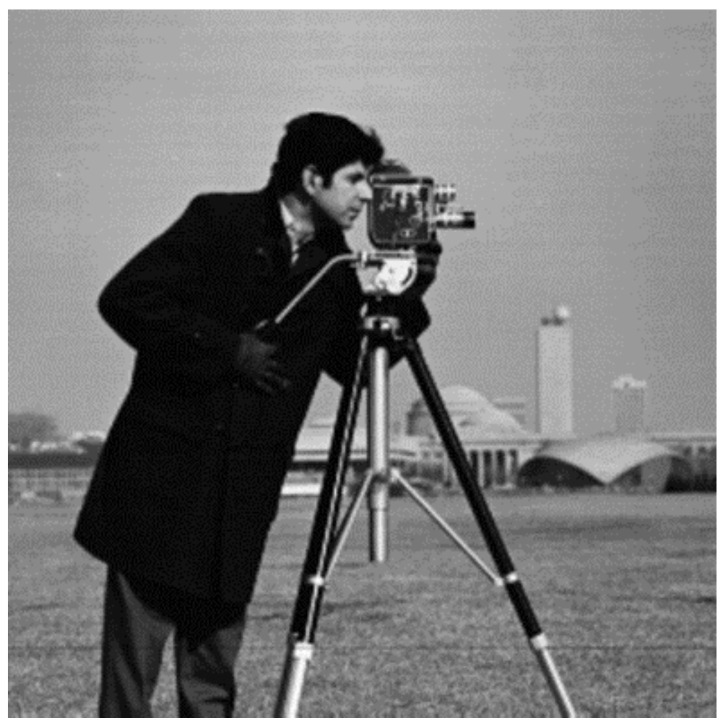
Cameraman.

**Figure 7:**
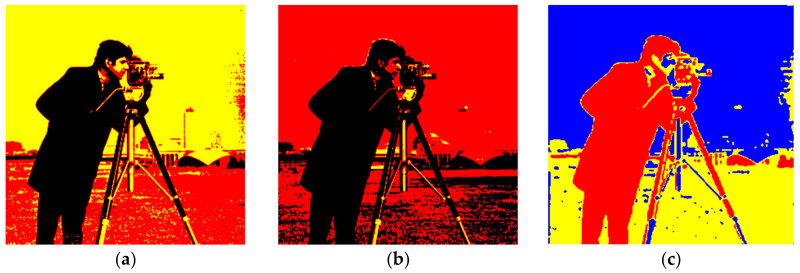
*m* = 2 and *c* = 3 (**a**) Otsu, T = 0.125 s. (**b**) Kapur, T = 0.114 s. (**c**) FCM, T = 0.891 s.

**Figure 8:**
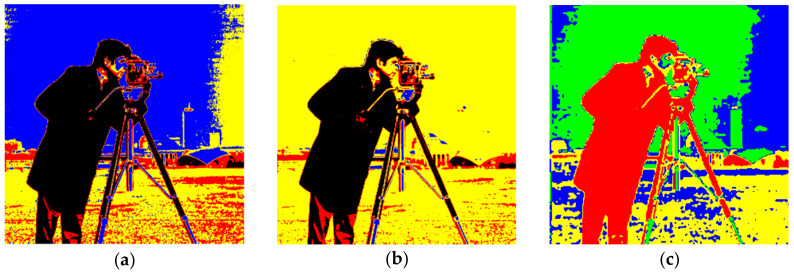
*m* = 3 and *c* = 4 (**a**) Otsu, T = 0.472 s. (**b**) Kapur, T = 0.321 s. (**c**) FCM, T = 4.310 s.

The recommended technique works with both gray scale and color images. For example, when a gray scale image is considered with a single threshold, either S_0_ or S_7_ in Table 1 and the color space are triggered. That means only two clusters will be activated: binarization. Consequently, only diagonal sub-cubes in the color space will be employed with a gray scale image for a multilevel threshold. The cameraman image shown in Figure 6 was initially tested in experiments where a 64-bit personal computer, 2.20 GHz was employed. As the binarization result of the cameraman image was already known, multilevel thresholds were estimated as shown in Table 2. During the experiments, the true values of the thresholds were primarily found by means of a linear search algorithm, which is the slowest one available. The thresholds were then estimated with the FOA and PSO algorithms to compare the speed and accuracy. According to Equation (5), the maximum number of clusters depends on the number of thresholds. Therefore, the cameraman image was also clustered with the FCM algorithm where the cluster number was set as compatible with Table 2. Thus, the speed of the proposed algorithm was also compared with the well-known clustering algorithm in image segmentation applications. The segmented images obtained with the Otsu, Kapur, and FCM algorithms are as shown in Figure 7a–c, respectively. On the other hand, when the threshold number, *m*, was selected as 3, the results obtained with the Otsu, Kapur, and FCM algorithms are shown in Figure 8a–c, respectively. As can be seen, the proposed multilevel thresholding algorithm produced reasonable results faster than conventional FCM algorithms. 

**Figure 9:**
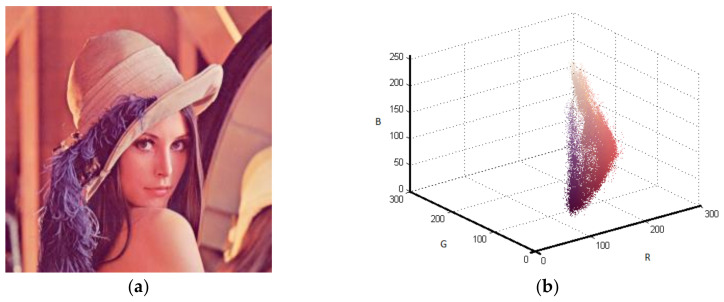
Lena (**a**) Original (**b**) Color distribution.

**Figure 10:**
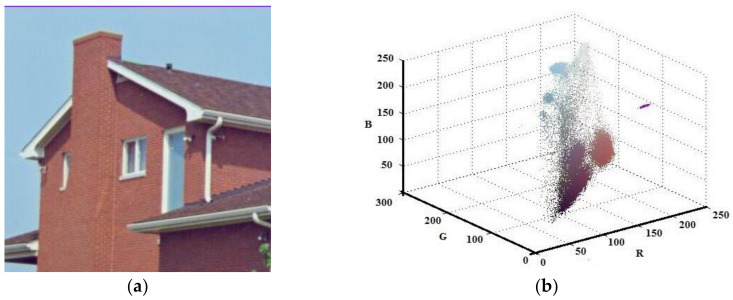
House (**a**) Original (**b**) Color distribution.

**Figure 11:**
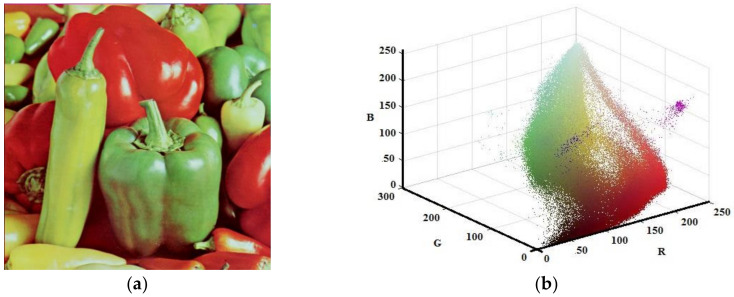
Pepper (**a**) Original (**b**) Color distribution.

Although the developed algorithm has worked for gray scale image, the main contribution of this study has been on color images. Thus, the Lena, House, and Pepper images shown in Figure 9a, Figure 10a, and Figure 11a, respectively, were tested with the suggested algorithm. Color distribution of the test images in the color space are given in Figure 9b, Figure 10b, and Figure 11b, respectively. Additionally, thresholds obtained with the proposed algorithm are shown in Table 3, Table 4 and Table 5, respectively.

Although Equation (5) gives the maximum number of clusters that could be filled in the color space, it is not guaranteed that all sub-cubes or prisms created in the color space will be filled with pixels. It depends on the distribution of pixels in the color space and the volume of the prisms. If the threshold numbers increase, the volume of the sub-prisms will decrease. On the other hand, if the volumes of the prisms are large, the probability occupied by the prism will increase. However, homogeneities of regions are reduced. During experiments, test images were filtered with a median filter in order to eliminate the creation of regions with a single or few pixels.

**Figure 12:**
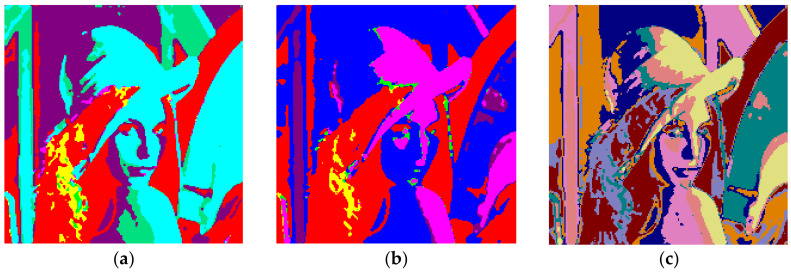
Lena, *m* = 1 (**a**) Otsu, *c* = 8, T = 0.244 s. (**b**) Kapur, *c* = 6, T = 0.294 s. (**c**) FCM, *c* = 8, T = 3.410 s.

Figure 12 shows the results obtained with Lena and *m* = 1. The total number of clusters filled with the Otsu algorithm was eight, as shown in Figure 12a, and the computation time (T) was 0.244 s. On the other hand, six prisms were occupied with the Kapur technique, as shown in Figure 12b, and the computation time (T) was 0.294 s. The performance of the FCM algorithm when the cluster number, *c,* was set to 8, is shown in Figure 12c and the computation time (T) was 3.410 s. As can be seen, the proposed method is faster than FCM. 

**Figure 13:**
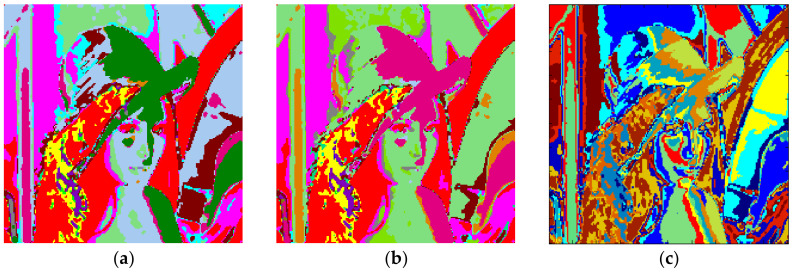
Lena, *m* = 2 (**a**) Otsu, *c* = 19, T = 1.141 s. (**b**) Kapur, *c* = 18, T = 1.022 s. (**c**) FCM, *c* = 19, T = 7.141 s.

**Figure 14:**
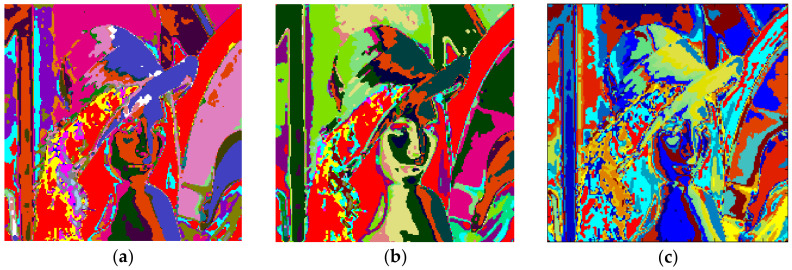
Lena, *m* = 3 (**a**) Otsu, *c* = 32, T = 1.163 s. (**b**) Kapur, *c* = 30, T = 1.133 s. (**c**) FCM, *c* = 32, T = 11.506 s.

The clustering performance of the algorithm with Lena and *m* = 2 is shown in Figure 13. Although the maximum number of clusters to be created was 27, only 19 of them were occupied with Otsu as shown in Figure 13a. Also, when the Kapur method was used, 18 clusters were completed as shown in Figure 13b. Moreover, the FCM algorithm produced results in 7.141 s for 19 clusters as in Figure 13c. The final experiment with Lena was done with *m* = 3. Figure 14a shows the results obtained with Otsu, while the performance of the Kapur principle is given in Figure 14b. Furthermore, the FCM algorithm produced the results shown in Figure 14c in 11.506 s, while the proposed algorithm completed the segmentation process within 1.163 s. 

**Figure 15:**
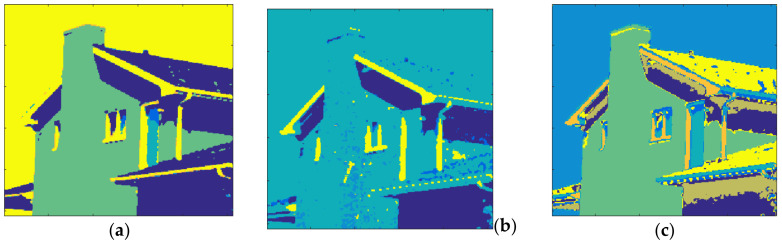
House, *m* = 1 (**a**) Otsu, *c* = 8, T = 0.205 s. (**b**) Kapur *c* = 6, T = 0.294 s. (**c**) FCM, *c* = 8, T = 4.121 s.

Figure 15 shows the results obtained with House and *m* = 1. The total number of clusters filled with the Otsu algorithm was eight as shown in Figure 15a and the computation time (T) was 0.205 s. On the other hand, six prisms were occupied with the Kapur technique as shown in Figure 15b and the computation time (T) was 0.294 s. The performance of the FCM algorithm with which the cluster number, *c*, was set to 8, is shown in Figure 15c and the computation time (T) was 4.121 s. As can be seen, the proposed method is faster than FCM.

**Figure 16:**
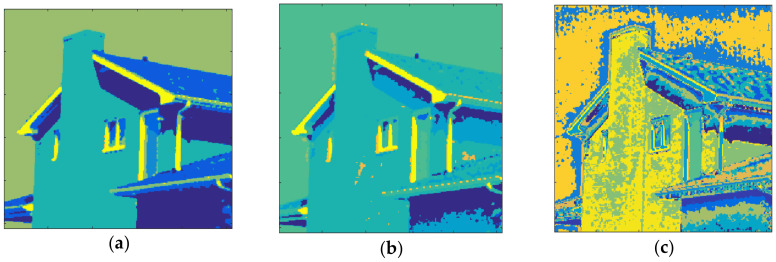
House, *m* = 2 (**a**) Otsu, *c* = 19, T = 1.321 s. (**b**) Kapur *c* = 15, T = 1.531 s. (**c**) FCM, *c* = 19, T = 11.260 s.

**Figure 17:**
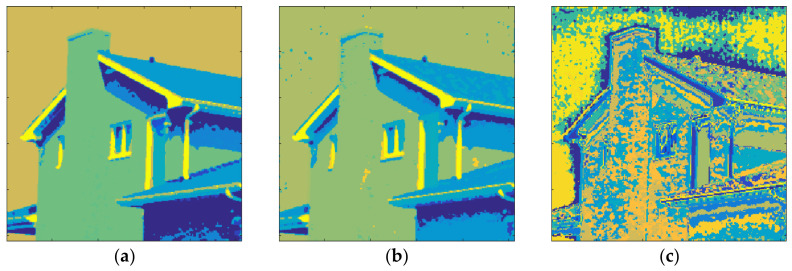
House, *m* = 3 (**a**) Otsu, *c* = 36, T = 1.293 s. (**b**) Kapur, *c* = 26, T = 1.237 s. (**c**) FCM, *c* = 36, T = 20.289 s.

The clustering performance of the algorithm with House and *m* = 2 is shown in Figure 16. Although the maximum number of clusters to be created was 27, only 19 of them were occupied with Otsu, as shown in Figure 16a. Also, when the Kapur method was used, 15 clusters were completed, as shown in Figure 16b. Furthermore, the FCM algorithm produced results for 19 clusters in 11.260 s, as in Figure 16c. The final experiment with House was done with *m* = 3. Figure 17a shows the results obtained with Otsu while the performance of the Kapur principle is given in Figure 17b. Furthermore, the FCM algorithm produced the result shown in Figure 17c in 20.289 s, while the proposed algorithm completed the segmentation process within 1.293 s.

**Figure 18:**
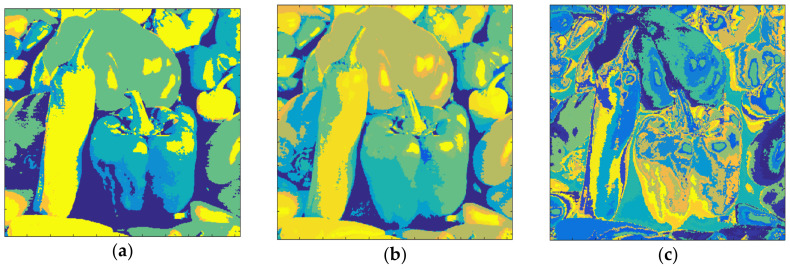
Pepper, *m* = 1 (**a**) Otsu, *c* = 8, T = 0.324 s. (**b**) Kapur, *c* = 8, T = 0.397 s. (**c**) FCM, *c* = 8, T = 5.184 s.

Figure 18 shows the results obtained with Pepper and *m* = 1. The total number of clusters filled with the Otsu algorithm was eight, as shown in Figure 18a, and the computation time (T) was 0.324 s. On the other hand, eight prisms were occupied with the Kapur technique, as shown in Figure 18b, and the computation time (T) was 0.397 s. The performance of the FCM algorithm when the cluster number, *c*, was set to 8, is shown in Figure 18c and the computation time (T) was 5.184 s. As can be seen, the suggested method is faster than FCM. 

**Figure 19:**
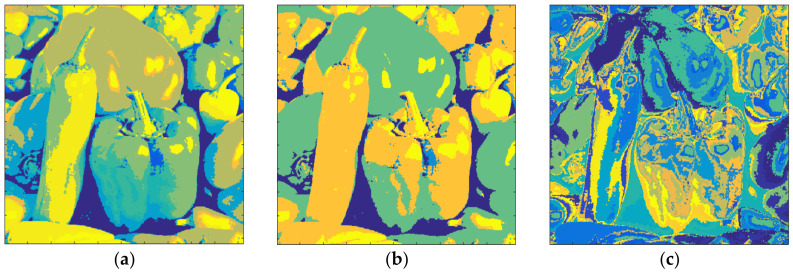
Pepper, *m* = 2 (**a**) Otsu, *c* = 26, T = 1.413 s. (**b**) Kapur, *c* = 22, T = 1.743 s. (**c**) FCM, *c* = 26, T = 13.152 s.

**Figure 20:**
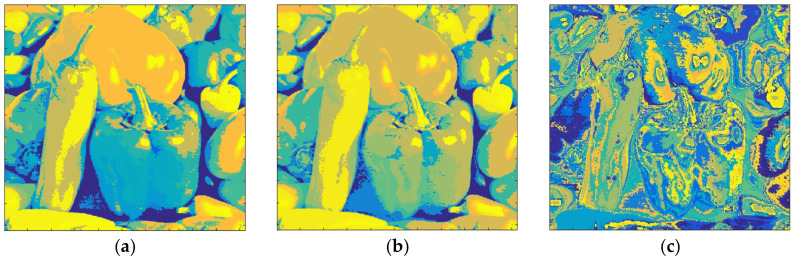
Pepper, *m* = 3 (**a**) Otsu, *c* = 52, T = 1.582 s. (**b**) Kapur, *c* = 48, T = 2.477 s. (**c**) FCM, *c* = 52, T = 22.178 s.

The clustering performance of the algorithm with Pepper and *m* = 2 is shown in Figure 19. Although the maximum number of clusters to be created was 27, only 26 of them were occupied with Otsu, as shown in Figure 19a. Also, when the Kapur method was used, 22 clusters were completed, as shown in Figure 19b. Moreover, the FCM algorithm created results in 13.152 s for 26 clusters as in Figure 19c. The final experiment with Pepper was done with *m* = 3. Figure 20a shows the outputs with Otsu while the performance of the Kapur approach is given in Figure 20b. Furthermore, the FCM algorithm created the result shown in Figure 20c in 22.178 s, while the proposed algorithm completed the segmentation process within 1.582 s. 

The performance of the segmentation algorithms was tested with the evolution function defined in Equation (7). Threshold numbers, *F*-values, and the time parameters of Lena, House, and Pepper are given in Table 6, Table 7 and Table 8, respectively. As can be seen, the difference between *F*-values, FCM and the proposed algorithm is insignificant. On the other hand, the developed algorithm is faster than conventional FCM. The increase in computation time with the number of clusters or thresholds in FCM is higher than that of the proposed method. For example, in Table 8, when the cluster number is 8, the computation time for the proposed method is 0.397 s, whereas the computation time for FCM is 5.184 s. Thus, the proposed algorithm is 13 times faster than FCM.

The experimental results show that the color images are clustered faster than with the FCM algorithm. Only the threshold values for each channel were used in the developed algorithm. The parameter *m* in Equation (5) determines the maximum number of sub-prisms. Nevertheless, it is apparent from Figure 9, Figure10 and Figure 11 that there are empty areas in the color space that are not occupied. Although the specific clusters are created for such areas, they will not be used. Consequently, the distribution of pixels in the color space has a critical importance. Furthermore, the volume of the sub-prisms determines the homogeneity of the clusters created. The less volume is formed, the more homogeneous the created cluster. Therefore, a larger value of *m* produces smaller cubes. Nevertheless, it is observed that when *m* is 3, the maximum number of clusters will be 64, which could generally be sufficient for image segmentation applications. Moreover, the suggested method uses the one-dimensional histogram of the image rather than the two-dimensional image space. Thus, the developed algorithm is independent of the image size. 

## 4. Conclusions

A new segmentation algorithm for color images that is fast and fully automatic was developed. The devised algorithm requires only a single parameter and is free of iteration, unlike FCM. The integration of multilevel thresholding techniques with color space is possible to obtain sub-prisms and significant clusters in color images. In other words, it was shown that multilevel thresholding techniques could be used for color images. If the number of thresholds increases, the homogeneity of the clusters increases in the color image. Accordingly, the fundamental criteria of image clustering processes could be controlled with the number of thresholds. Additionally, as the individual histograms of color components are used in the proposed procedure, the time required for clustering is not dependent on the size of the image to be segmented. This contribution is also important for large scale images and real-time processing. Also it was shown that color image thresholding is possible by means of a color space partition. In this study, the number of thresholds used in each channel was the same. However, a different number of thresholds for each channel could be tested in future investigation. 

## Figures and Tables

**Table 1:** Color space partition with single threshold.

Class Label	Partition Rules	Binary Code
S_0_	if (R <= T_r,1_ & G <= T_g,1_ & B <= T_b,1_)	000
S_1_	if (R <= T_r,1_ & G <= T_g,1_ & B >= T_b,1_)	001
S_2_	if (R <= T_r,1_ & G >= T_g,1_ & B <= T_b,1_)	010
S_3_	if (R <= T_r,1_ & G >= T_g,1_ & B >= T_b,1_)	011
S_4_	if (R >= T_r,1_ & G <= T_g,1_ & B <= T_b,1_)	100
S_5_	if (R >= T_r,1_ & G <= T_g,1_ & B >= T_b,1_)	101
S_6_	if (R >= T_r,1_ & G >= T_g,1_ & B <= T_b,1_)	110
S_7_	if (R >= T_r,1_ & G >= T_g,1_ & B >= T_b,1_)	111

**Table 2:** Threshold for cameraman image with different methods.

	Otsu					Kapur					
	**Time (s)**	***t*_1_**		***t*_2_**		**Time (s)**		***t*_1_**		***t*_2_**	
**Linear**	0.600	71		145		0.836		124		192	
**FOA**	0.125	70		144		0.114		125		191	
**PSO**	0.360	71		145		0.517		124		192	
**Cluster: c**	3					3					
		***t*_1_**	***t*_2_**		***t*_3_**		***t*_1_**		***t*_2_**		***t*_3_**
**Linear**	164.713	46	102		150	243.758	45		103		192
**FOA**	0.472	48	104		150	0.321	45		103		192
**PSO**	0.777	46	102		150	0.628	45		103		192
**Cluster: c**	4					4					

**Table 3:** Thresholds for Lena image with different methods.

	Otsu												Kapur													
	Linear				FOA				PSO				Linear				FOA						PSO			
	***t*_1_**				***t*_1_**				***t*_1_**				***t*_1_**				***t*_1_**					***t*_1_**				
**R**	162				160				160				167				168						168			
**G**	102				103				103				141				140						140			
**B**	112				110				110				131				131						131			
**Time (s)**	0.008				0.244				0.244				0.029				0.294						0.333			
**Cluster: c**					8								6													
	***t*_1_**		***t*_2_**		***t*_1_**		***t*_2_**		***t*_1_**		***t*_2_**		***t*_1_**		***t*_2_**		***t*_1_**			***t*_2_**			***t*_1_**		***t*_2_**	
**R**	139		197		135		192		138		196		134		191		135			192			134		191	
**G**	79		146		83		152		79		146		82		151		83			150			82		151	
**B**	96		140		99		145		96		140		98		144		100			145			98		144	
**Time (s)**	5.572				1.141				1.567				6.628				1.022						2.310			
**Cluster: c**					19								18													
	***t*_1_**	***t*_2_**		***t*_3_**	***t*_1_**	***t*_2_**		***t*_3_**	***t*_1_**	***t*_2_**		***t*_3_**	***t*_1_**	***t*_2_**		***t*_3_**		***t*_1_**	***t*_2_**		***t*_3_**		***t*_1_**	***t*_2_**		***t*_3_**
**R**	129	178		213	132	180		213	132	180		213	116	159		203		117	160		203		116	161		203
**G**	58	107		160	61	109		161	61	109		161	61	110		161		60	110		160		61	110		161
**B**	84	111		147	84	110		145	84	110		145	93	132		170		92	132		169		93	132		170
**Time (s)**	512.833				1.163				1.818				676.992					1.133					2.630			
**Cluster: c**	34												29													

**Table 4:** Thresholds for House with different methods.

	Otsu												Kapur												
	Linear				FOA				PSO				Linear				FOA					PSO			
	***t*_1_**				***t*_1_**				***t*_1_**				***t*_1_**				***t*_1_**					***t*_1_**			
**R**	135				135				135				178				178					178			
**G**	144				144				144				88				89					88			
**B**	155				155				155				89				89					88			
**Time (s)**	0.007				0.205				0.194				0.029				0.294					0.333			
**Cluster: c**	8												6												
	***t*_1_**		***t*_2_**		***t*_1_**		***t*_2_**		***t*_1_**		***t*_2_**		***t*_1_**		***t*_2_**		***t*_1_**			***t*_2_**		***t*_1_**		***t*_2_**	
**R**	133		187		134		188		134		188		96		178		97			179		97		179	
**G**	80		151		81		152		81		152		88		202		89			203		89		203	
**B**	127		192		128		192		128		193		117		184		118			185		118		185	
**Time (s)**	5.371				1.321				1.242				5.113				1.531					1.821			
**Cluster: c**	19												15												
	***t*_1_**	***t*_2_**		***t*_3_**	***t*_1_**	***t*_2_**		***t*_3_**	***t*_1_**	***t*_2_**		***t*_3_**	***t*_1_**	***t*_2_**		***t*_3_**		***t*_1_**	***t*_2_**		***t*_3_**	***t*_1_**	***t*_2_**		***t*_3_**
**R**	104	142		188	104	142		188	104	142		188	89	153		180		89	153		180	89	153		180
**G**	81	131		178	80	130		178	80	130		178	55	97		205		55	97		205	55	97		205
**B**	87	133		194	87	133		194	87	133		194	117	164		212		118	165		213	118	165		213
**Time (s)**	492.257				1.293				1.291				552.715					1.237				2.011			
**Cluster: c**	36												26												

**Table 5:** Thresholds for Pepper with different methods.

	Otsu												Kapur												
	Linear				FOA				PSO				Linear				FOA					PSO			
	***t*_1_**				***t*_1_**				***t*_1_**				***t*_1_**				***t*_1_**					***t*_1_**			
**R**	146				146				146				101				101					101			
**G**	111				111				111				129				129					129			
**B**	72				72				72				114				114					114			
**Time (s)**	0.007				0.324				0.313				0.031				0.397					0.322			
**Cluster: c**	8												8												
	***t*_1_**		***t*_2_**		***t*_1_**		***t*_2_**		***t*_1_**		***t*_2_**		***t*_1_**		***t*_2_**		***t*_1_**			***t*_2_**		***t*_1_**		***t*_2_**	
**R**	99		161		99		161		99		161		93		158		93			158		93		158	
**G**	80		159		80		159		80		159		68		151		68			151		68		151	
**B**	59		128		59		128		59		128		107		166		107			166		107		166	
**Time (s)**	5.534				1.413				1.435				5.783				1.743					1.629			
**Cluster: c**	26												22												
	***t*_1_**	***t*_2_**		***t*_3_**	***t*_1_**	***t*_2_**		***t*_3_**	***t*_1_**	***t*_2_**		***t*_3_**	***t*_1_**	***t*_2_**		***t*_3_**		***t*_1_**	***t*_2_**		***t*_3_**	***t*_1_**	***t*_2_**		***t*_3_**
**R**	89	137		177	89	137		177	89	137		177	61	105		163		61	105		163	61	105		163
**G**	36	99		168	36	99		168	36	99		168	65	120		172		65	120		172	65	120		172
**B**	29	68		131	29	68		131	29	68		131	62	115		169		62	115		169	62	115		169
**Time (s)**	478.563				1.582				1.512				529.054					2.477				2.392			
**Cluster: c**	52												48												

**Table 6:** Quantitative evaluation of Lena.

Number of Threshold/Cluster	Method	F	Time, T (s)
*m* = 1/*c* = 8	Otsu	0.00000251	0.244
*m* = 1/*c* = 6	Kapur	0.00000375	0.294
*c* = 8	FCM	0.00000237	3.410
*m* = 2/*c* = 19	Otsu	0.00001364	1.141
*m* = 2/*c* = 18	Kapur	0.00001348	1.022
*c* = 19	FCM	0.00000654	7.141
*m* = 3/*c* = 32	Otsu	0.00002830	1.163
*m* = 3/*c* = 30	Kapur	0.00002332	1.133
*c* = 32	FCM	0.00000609	11.506

**Table 7:** Quantitative evaluation of House.

Number of Threshold/Cluster	Method	F	Time, T (s)
*m* = 1/*c* = 8	Otsu	0.00000819	0.205
*m* = 1/*c* = 6	Kapur	0.00000620	0.294
*c* = 8	FCM	0.00000046	4.121
*m* = 2/*c* = 19	Otsu	0.00001489	1.321
*m* = 2/*c* = 15	Kapur	0.00001248	1.531
*c* = 19	FCM	0.00000278	11.260
*m* = 3/*c* = 36	Otsu	0.00004402	1.293
*m* = 3/*c* = 26	Kapur	0.00005342	1.237
*c* = 36	FCM	0.00000749	20.289

**Table 8:** Quantitative evaluation of Pepper.

Number of Threshold/Cluster	Method	F	Time, T (s)
*m* = 1/*c* = 8	Otsu	0.00000386	0.324
*m* = 1/*c* = 8	Kapur	0.00000508	0.397
*c* = 8	FCM	0.00000014	5.184
*m* = 2/*c* = 26	Otsu	0.00003469	1.413
*m* = 2/*c* = 22	Kapur	0.00000592	1.743
*c* = 26	FCM	0.00000097	13.152
*m* = 3/*c* = 52	Otsu	0.00003018	1.582
*m* = 3/*c* = 48	Kapur	0.00002154	2.477
*c* = 52	FCM	0.00000188	22.178
